# Extract from Maize (*Zea mays* L.): Antibacterial Activity of DIMBOA and Its Derivatives against *Ralstonia solanacearum*

**DOI:** 10.3390/molecules21101397

**Published:** 2016-10-19

**Authors:** Bing Guo, Yongqiang Zhang, Shili Li, Ting Lai, Liang Yang, Juanni Chen, Wei Ding

**Affiliations:** Laboratory of Natural Products Pesticides, College of Plant Protection, Southwest University, Chongqing 400715, China; guobing425@163.com (B.G.); zyqiang@swu.edu.cn (Y.Z.); lsl203lst@163.com (S.L.); laiting93@163.com (T.L.); ylwzling@163.com (L.Y.); chenhuanni521@126.com (J.C.)

**Keywords:** DIMBOA, *Ralstonia solanacearum*, antibacterial activity, biofilm inhibition, bacterial wilt

## Abstract

Many cereals accumulate hydroxamic acids involved in defense of plant against various fungi, bacteria, and insects. 2,4-dihydroxy-7-methoxy-1,4-benzoxazine-3-one, commonly known as DIMBOA, is one of the principal cyclic hydroxamic acids in aqueous extracts of maize. The aim of this study was to evaluate the antibacterial activity of the isolated DIMBOA and its derivatives 2-benzoxazolinone (BOA), 6-chloro-2-benzoxazolinone (CDHB), and 2-mercaptobenzothiazole (MBT) against *Ralstonia solanacearum.* MBT showed the strongest antibacterial activity, followed by CDHB and DIMBOA, with minimum inhibitory concentrations (MICs) of 50, 100 and 200 mg/L, respectively, better than the BOA with 300 mg/L. These compounds also significantly affect bacterial growth, reduce biofilm formation, and inhibit swarming motility within 24 h. This paper is the first to report the anti-*R. solanacearum* activity of DIMBOA from *Z. mays*. The bioassay and pot experiment results suggested that DIMBOA and its derivatives exhibit potential as a new matrix structure of designing target bactericide or elicitor for controlling tobacco bacterial wilt. Further studies must evaluate the efficacy of DIMBOA and its derivatives in controlling bacterial wilt under natural field conditions where low inoculum concentrations exist.

## 1. Introduction

Bacterial wilt caused by *Ralstonia solanacearum* is one of the most significant diseases of tobacco in southwestern China. The principal widespread hosts of *R. solanacearum* are the *Solanaceae* and *Musaceae* families of potato (*Solanum tuberosum*), tobacco (*Nicotiana tabacum*), and tomato (*Lycopersicon esculentum*) [1]. Integrated pest management strategies developed include the use of resistant cultivars, healthy transplants, and crop rotation with non-host cover crops [2]. Although crop rotation with non-host crops may suppress soil-borne populations of the pathogen [3,4], further evaluation is necessary for the biological secondary metabolites of rotation crops.

The benzoxazinoids derivatives are a group of cyclic hydroxamic acids which are found prevalently in the members of family *Poaceae*. In the *Poaceae* family, these have been reported from *Secale cereale* L., *Triticum aestivum* L. and *Zea mays* L. [5]. The benzoxazinoid derivatives were discovered in Nature in the 1950s and have been of significant scientific interest in nutrition and pharmaceutics during the past decade [6]. The group of chemical compounds named benzoxazinoid derivatives is subdivided into hydroxamic acids (Hx), lactams, benzoxazolinones, and methyl derivatives of the hydroxamic acids. Benzoxazinoid hydroxamic acids have been reported that exhibit phytotoxic activities, play a significant role in plant defense against fungi, bacteria, and insects, and participate in allelopathy mechanisms [5,7,8,9]. Benzoxazinoid hydroxamic acids are implicated in below-ground defense, where they exert allelochemical or antimicrobial activities. Crop rotation or intercropping is an effective measure used to control pepper soil-borne diseases. The root exudates of maize can attract zoospores to the root tip, thereby quickly stopping the activity of these spores and encysting them into cysto-spores [10]. However, most studies have focused on benzoxazinoid compounds serve as an important factor in the host plant’s resistance against fungal diseases and insects, and act as potent allelochemicals. There are few studies explore the antibacterial activity of benzoxazinoid hydroxamic acids against soil-borne pathogens, especially *R. solanacearum.*

Benzoxazinoid hydroxamic acids are widely used in agro-technology as substitutes or structural models to prepare new pesticides [11]. Epidemiological and in vitro studies reported that benzoxazinoid hydroxamic acids elicited health-protecting effects, such as anti-allergic, anti-microbial, anti-inflammatory, anticancer, and antidepressant [6]. Previous studies demonstrated that some hydroxamic acids possess strong antioxidant activity, i.e., HBOA derived from *Acanthus hirsutus* water extract [12]. Some plant phenolic compounds and their derivatives (e.g., *p*-coumaric acid, benzoic acid, *trans*-4-hydroxycinnamic acid) also inhibit T3SS in plant pathogens, such as *Erwinia amylovora* and *Dickeya dadantii* [13,14,15]. Cereals, particularly wheat, can produce various benzoxazinone derivates, such as hydroxamic acids (e.g., DIMBOA) or benzoxazolinones (e.g., MBOA) with high allelopathic activity. The present study aims to isolate the cyclic hydroxamic acid 2,4-dihydroxy-7-methoxy-2*H*-1,4-benzoxazin-3(4*H*)-one (DIMBOA)—from maize tissues (*Zea mays* L.) —and evaluate its antibacterial activity against *R. solanacearum*.

## 2. Results

### 2.1. Determination of DIMBOA Antibacterial Activity

**Figure 1:**
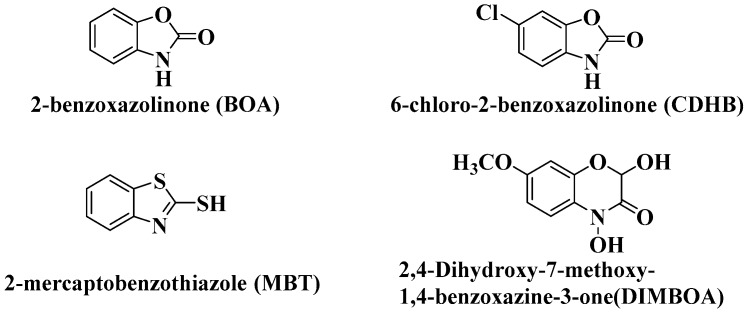
The structure of DIMBOA and its derivatives.

**Figure 2:**
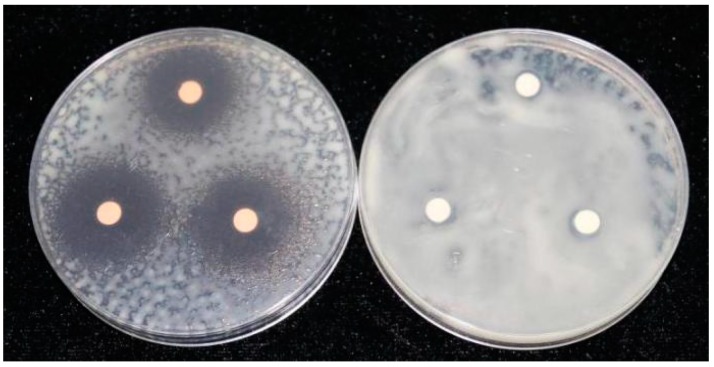
Effect of DIMBOA and 1% DMSO on the growth of *R. solanacearum* at the concentration of 300 μg/disc.

In this study, 2,4-dihydroxy-7-methoxy-1,4-benzoxazine-3-one (DIMBOA) and its derivatives 2-benzoxazolinone (BOA), 6-chloro-2-benzoxazolinone (CDHB), and 2-mercaptobenzothiazole (MBT) efficiently inhibited the growth of *R. solanacearum* (Table 1). DIMBOA possesses moderate antibacterial activity against *R. solanacearum*. Antibacterial activity was enhanced with increasing concentration of DIMBOA and its derivatives, and the highest activity was obtained from CDHB and DIMBOA at 300 μg/disc. Relative antibacterial activity showed differences correlated with the concentration of DIMBOA and its derivatives. The results also showed that hydrosulphonyl and chlorine substituents (Figure 1) enhanced the bactericidal activity against *R. solanacearum*. The solvent DMSO did not affect the growth of *R. solanacearum* on the plates after 24 h (Figure 2).

### 2.2. MIC and MBC of DIMBOA and Its Derivatives against R. solanacearum

The MIC and MBC of DIMBOA against *R. solanacearum* were measured using typical micro-dilution method. As shown in Table 2, MBT was the most effective compound against *R. solanacearum*, followed by CDHB, DIMBOA, and 2-benzoxazolione. The MIC of MBT was 50 mg/L, which is lower than the value of the extracted compound DIMBOA (200 mg/L). This finding indicates that the inhibitory efficiency of MBT is fourfold higher than that of DIMBOA. The MICs of CDHB and 2-benzoxazolione were 100 and 300 mg/L, respectively. The minimum bactericidal concentrations (MBCs) of DIMBOA and its derivatives on *R. solanacearum* were defined as their lowest concentration that prevents the growth of bacteria after sub-culture on agar media. The MBCs of CDHB, DIMBOA, and MBT against *R. solanacearum* were 500, 400, and 500 mg/L, respectively. The MBCs of 2-benzoxazolione was likely related to its degradation which exceeded the test concentration of 1000 mg/L. Hence, DIMBOA and its derivatives are antibiotics eliciting modest toxicity (50–500 mg/L) to *R. solanacearum*.

To evaluate the quantitative correlation between the concentration and the antibacterial activity of DIMBOA and its derivatives against *R. solanacearum*, we also simulated the linear relationship between the logarithmic value of DIMBOA and its derivatives concentration. The probability value of the corrected antibacterial rate was also determined based on the microplate reader test. After 24-h incubation, the growth of *R. solanacearum* entered the stable phase. As shown in Table 3, the IC_50_ values of BOA, CDHB, MBT, and DIMBOA were 208.92, 29.64, 8.24, and 58.55 mg/L, respectively. However, the IC_90_ value is several-fold higher than IC_50_, approximately 4.33, 17.62, 20.39, 6.25 times, respectively.

### 2.3. DIMBOA and Its Derivatives Inhibit the Growth of R. solanacearum

**Figure 3:**
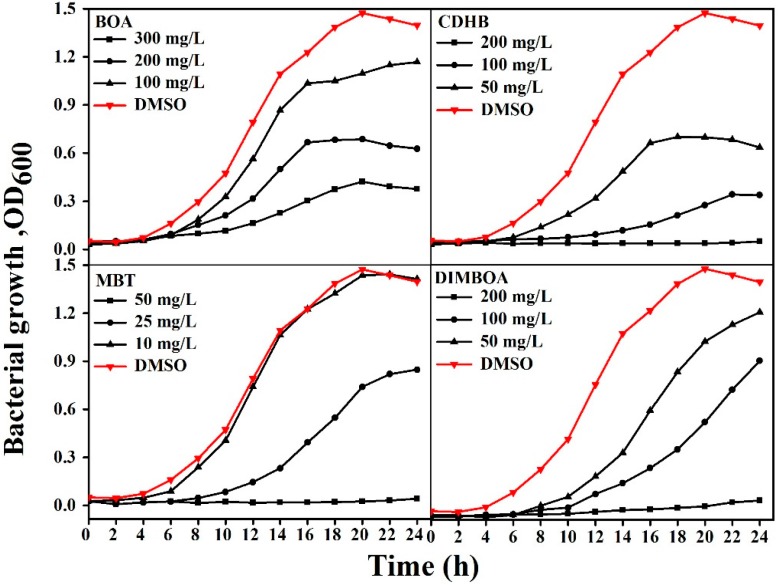
The effect of DIMBOA and its derivatives at different concentrations on the growth curves of *R. solanacearum*.

According to the MICs, we then set three concentrations to further investigate the effect of DIMBOA and its derivatives at different concentrations on the growth curves of *R. solanacearum*. The cell growth of *R. solanacearum* was inhibited by DIMBOA and its derivatives inordinately. BOA and CDHB significantly inhibited the growth of *R. solanacearum* at 300 and 100 mg/L, respectively. CDHB, MBT, and DIMBOA at concentrations of 200, 50 and 200 mg/L absolutely stopped *R. solanacearum* growth throughout the test. To conclude, the antibacterial activity of DIMBOA and its derivatives against *R. solanacearum* increased with dosage (Figure 3).

### 2.4. DIMBOA and Its Derivatives Reduce Biofilm Formation of R. solanacearum

**Figure 4:**
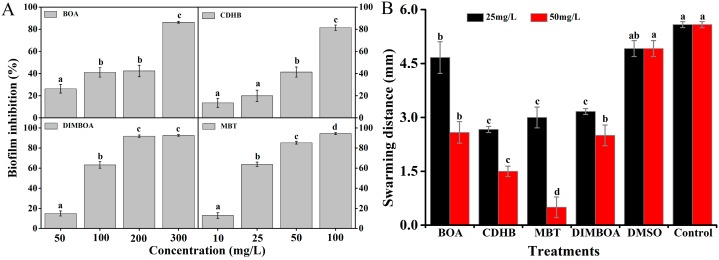
Effects of DIMBOA and its derivatives on biofilm formation (**A**) and swarming motility(**B**) of *R. solanacearum* after 24 h.(**A**) Biofilm inhibition (%) was quantified after treatment with different concentrations of DIMBOA and its derivatives at 30 ± 1 °C for 24 h in the 96-well plates; (**B**) The swarming diameter was measured in both the vertical and horizontal direction on each plate after incubation at 30 ± 1 °C for 24 h. The mean value in both directions was calculated. The diameter represents the average of triplicate plates. The assays were independently repeated three times. The error bars indicate the standard error of the mean from three replicates. Lower case letters indicate significant differences according to Duncan’s test (*p* < 0.05).

As shown in Figure 4A, DIMBOA and its derivatives significantly reduced the biofilm formation of *R. solanacearum*; CDHB especially reduced biofilm formation by 81.42% at a concentration of 100 mg/L compared with 41.11% by BOA treatment. MBT and DIMBOA also significantly reduced the biofilm formation with an inhibitory rate of 60% or higher, especially the concentrations up to 25 and 100 mg/L, respectively. The preliminary results showed that MBT exhibited the highest inhibitory ability on *R. solanacearum* biofilm formation, followed by CDHB, DIMBOA, and BOA. The inhibitory activity of DIMBOA and its derivatives were concentration dependent. The motility of *R. solanacearum* has been demonstrated at a molecular level to be important in biofilm formation and pathogenicity [16]. Thus, the swarming motility under the treatment of DIMBOA and its derivatives was also investigated. The results indicated that DIMBOA and its derivatives could significantly inhibit swarming motility at concentrations 25 and 50 mg/L after 24 h (Figure 4B). At 25 mg/L, the diameters of the migration zone of CDHB and MBT were decreased by 1.32- and 2.50-fold compared with DMSO. 

###  2.5. DIMBOA and Its Derivatives Enhanced the Resistance of Tobacco Seedlings to R. solanacearum

**Figure 5:**
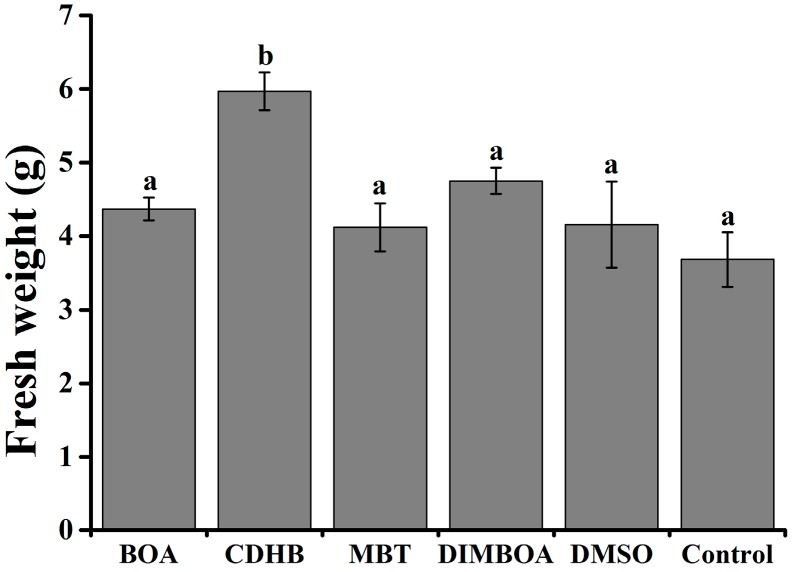
Effects of DIMBOA and its derivatives at the minimum inhibitory concentrations on tobacco fresh weight. The 10-mL solution of DIMBOA and its derivatives (MICs) and DMSO (0.3%) were respectively irrigated into the rhizosphere of each 4-week-old tobacco seedling, then the fresh weight was measured after 7 days. The assays were independently repeated three times. BOA at concentrations of 300 mg/L; CDHB at concentrations of 100 mg/L; MBT at concentrations of 50 mg/L; DIMBOA at concentrations of 200 mg/L. The error bars indicate the standard error of the mean from three replicates. Lower case letters indicate significant differences according to Duncan’s test (*p* < 0.05).

**Figure 6:**
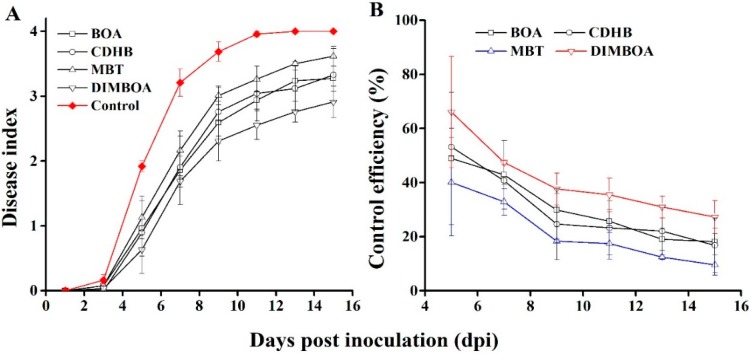
The disease index (**A**) and control efficiency **(B**) of DIMBOA and its derivatives on seedlings inoculated with *R. solanacearum*. The 10-mL solution of DIMBOA and its derivatives (10 mg/L) and DMSO (0.05%) were respectively irrigated into tobacco roots, then 10 mL of freshly cultured suspension of *R. solanacearum* (1 × 10^8^ CFU/mL) was inoculated to the rhizosphere of each 4-week-old tobacco plants which were treated with DIMBOA and its derivatives after 24 h. Symptoms were rated daily using a disease index scale of 0–4 (0, no symptoms appeared; 1, 1%–25% of leaves wilted; 2, 26%–50% of leaves wilted; 3, 51%–75% of leaves wilted; 4, 76%–100% of leaves wilted). Each point represents the average disease index of 20 plants. The DIMBOA and its derivatives treatment was significantly different from the DMSO treatment (*p* < 0.05; repeated-measures ANOVA). Similar results were observed in two other independent experiments.

DIMBOA and its derivatives at the concentration which were effective against *R. solanacearum* in vivo. Nevertheless, pot experiment demonstrated that these compounds have no toxic on tobacco seedlings (Figure 5), and low cytotoxicity on human cells [17]. All treatments indicate that average levels of fresh weight were higher than control. Unexpectedly, CDHB could promote the fresh weight significantly. In addition, previous reports demonstrated that DIMBOA plays an important role in the resistance of plants [18,19]. Therefore, the incidence of disease from 1 to 13 days after treatment with DIMBOA and its derivatives (10 mg/L) and control (0.05% DMSO) on tobacco roots was investigated. The application of DIMBOA and its derivatives did not provide an effective control of bacterial wilt incidence. However, bacterial wilt index in the treated plants was reduced compared with the control (Figure 6A). The control efficiency of DIMBOA and MBT were kept at the lowest levels of 27.24% and 9.55%, respectively, at the 15th day (Figure 6B). As a whole, DIMBOA showed the possibility of elicitors and reduced the disease index of bacterial wilt.

## 3. Discussion

During the past decades, agricultural control, chemical control, disease resistance breeding, and biological control methods were used as the traditional methods to control tobacco bacterial wilt, and exert certain control effects on tobacco bacterial wilt outbreaks and epidemics [1]. As the application of traditional agrochemicals has not proven very effective in controlling this soil-borne bacterium, agricultural control still represents the main control. Meanwhile, the application of corn rotation has been proven to have a considerable effect. However, much of the fundamental research underlying these discussions has been confined to soil nutrient, microbes [20,21], where the secondary metabolites of rotation crops are generally with fewer considerations. The glucosides of cyclic hydroxamic acids such as DIMBOA are present in some species of the *Poaceae*. In plants, these glucosides and are released as aglycones by the activity of enzyme α-glucosidase after plant injury (attack by insects, crushing of plant tissue or deterioration of plant tissue), and then to the corresponding benzoxazolinones by spontaneous re-arrangement [22]. The resulting compounds are inhibitory to fungi, and some pathogens of grasses are able to detoxify them [23]. 

Wheat, rye and maize are the only agricultural crops with known presence of hydroxamic acids [5]. DIMBOA and 2,4-dihydroxy-6,7-dimethoxy-l,4-benzoxazin-3-one (DIM_2_BOA) glucosides have been reported to occur in both the shoot and root of germinating maize, followed by their aglycones DIMBOA and DIM_2_BOA, and that these compounds, especially aglycones, attain a maximum level soon after the germination and then disappear gradually as the seeds after germinated 10 days [24,25].Therefore, DIMBOA was isolated from 7-day-old etiolated maize seedlings and characterized by UV, melting point apparatus, and NMR. Amounts of DIMBOA present in extracts were estimated by the external reference method of HPLC. Previous studies have indicated that DIMBOA has antioxidant and antifungal activity [26], antibacterial activity [18], and insects [27], and participate in allelopathy mechanisms [28]. However, the antibacterial potentials of DIMBOA and its derivatives against *R. solanacearum* was not investigated. Among the tested compounds, MBT exhibited the best antibacterial activity, followed by CDHB, and DIMBOA, which showed that hydrosulphonyl and chlorine substituents enhanced the bactericidal activity against *R. solanacearum*. Ever, efforts are being made for enhancing their phytotoxicity through modifications of steric and electronic features of the basic benzoxazinoid skeleton [29]. These modifications included the addition of halogen substituents to the aromatic ring. The result reported in this study showed that the MBC (500 mg/L) was 2.5 times the MIC (200 mg/L) for DIMBOA. It would then be developed as a new type of botanical antibiotic or a new antibacterial drug, when structural modification of DIMBOA as lead compound is fulfilled. DIMBOA and its derivatives at concentrations of up to 50 mg/L exhibit high anti-biofilm formation activity partially, and also significantly repressed the swarming motility of *R. solanacearum*, resulting in weaker pathogenicity. DIMBOA and its derivatives did not reduce bacterial wilt incidence on a susceptible cultivar Yunyan 87 under greenhouse conditions when inoculum concentrations up to 10^8^ CFU/mL were used in this study. 

Consequently, the efficacy of DIMBOA and its derivatives in controlling bacterial wilt on susceptible tobacco cultivars under natural field conditions where low inoculum concentrations exist deserves further studies. Meanwhile, further studies also need to be conducted before practical use of DIMBOA and its derivatives for management of bacterial wilt of tobacco can be recommended to farmers. For example, timing and dosage of DIMBOA and application should be optimized and standardized for achieving maximum efficacy and economic benefit in large-scale field experiments. Levels of DIMBOA exudation showed a statistically significant linear decline in aging plants, which is exuded in relatively high quantities from roots of young maize seedlings [30]. DIMBOA is prevalent in maize tissues and can also be secreted into rhizosphere soil by roots [31,32]. When released from maize or added to soil, DIMBOA is microbial transformed to MBOA [33,34]. DIMBOA and MBOA could affect the soil microbial community structure, even at a low concentration of 5 μg/g of dry soil, to their advantage through the change in fungi populations [35]. The effect of most allele-chemicals on microbial activity is dose-dependent. Interestingly, mycorrhization of maize was recently reported to boost DIMBOA production [19]. Since mycorrhization is known to cause major qualitative changes in rhizobacterial communities [36], it is possible that increased DIMBOA exudation from mycorrhizal roots contributes to this so-called mycorrhizosphere effect. It might be expected that DIMBOA exudation by seedlings must provide significant ecological benefits. Further studies should be done on the microorganisms in the rhizosphere of corn or other graminaceous crops rotation of tobacco, which would possibly be seriously injured by *R. solanacearum*. Next, utilization of root exudations and species diversity to alter soil microbial community presenting a potential opportunity for biocontrol of tobacco bacterial wilt.

## 4. Materials and Methods

### 4.1. Bacterial Strains and Inoculum Preparation

The *R. solanacearum* (phylotype I, race 1, biovar 3) tobacco strain was used in this study [37]. The bacterial pathogen was grown at 30 °C either in rich B medium [38]. The tobacco plants were inoculated with bacterial suspensions (1.0 × 10^8^ CFU/mL).

### 4.2. Preparation of DIMBOA and Its Derivatives

DIMBOA was isolated from maize (*Z. mays* L.) seedlings based on the procedure established by Larsen [39] and Li [11]. The purity of DIMBOA was determined by analytical HPLC, UV, melting point, NMR data (^1^H and ^13^C), and mass spectrum analyses, consistent with those previously reported for DIMBOA [40,41,42]. All chemicals were dissolved in dimethyl sulfoxide (DMSO), Three DIMBOA derivatives, namely, BOA, CDHB, MBT, were purchased from Tokyo Chemical Industry (Tokyo, Japan). BOA can be also easily prepared by synthesis, e.g., using the di-*tert*-butyl dicarbonate/DMAP method [43]. DIMBOA was extracted from the etiolated maize seedlings. The final concentration of DIMBOA and its derivatives in DMSO was 50 mg/mL.

### 4.3. Determination of DIMBOA Antibacterial Activity

The antibacterial activity of DIMBOA and its derivatives against *R. solanacearum* were individually tested in vitro through filter paper disc agar diffusion method with minor modifications [26]. A suspension (100 μL) containing 10^8^ CFU/mL *R. solanacearum* was inoculated directly onto each NA medium. Three sterile filter paper discs (6 mm diameter) were applied aseptically to the surface of the agar plates. The stock solution was diluted to 30, 20, 10, 5, and 1 mg/mL, and 10 μL dilutions were dropped on the plate. DMSO (10 mg/mL) was used as control. The plates were incubated at 30 ± 1 °C for 24 h. The diameters of the inhibition zones, except the 6 mm disc diameter, were measured. Experiments were performed in triplicate, and the results were reported as mean values.

### 4.4. MIC and MBC Assay

The minimum inhibitory concentration (MIC) and minimum bactericidal concentration (MBC) of DIMBOA and its derivatives on *R. solanacearum* were determined using typical microdilution method [44]. Bacterial cells were suspended in rich B medium diluted to 10^5^–10^6^ colony-forming units (CFU/mL) for experimental use. The concentrations of DIMBOA and its derivatives were adjusted to 25, 50, 100, 200, 300, 400, 500, 600, 700, 800, 900, and 1000 mg/L. Each hole of the 96-well polystyrene microtiter plate was supplemented with 199 μL of the mixture and 1 μL of triphenyl tetrazolium chloride (TTC) as indicator of *R. solanacearum* growth. The plate was incubated without shaking for 24 h at 30 ± 1 °C. The MICs of DIMBOA and its derivatives were defined as the lowest concentration of compounds at which no pink color appeared. Briefly, 100 μL of the mixtures from the plate was transferred directly onto agar media for sub-culturing at 30 ± 1 °C for 24 h. The MBC endpoint was defined as the lowest concentration of antibiotic at which no visible growth of the organism is observed. All assays were carried out at least three times in biological repeats [45].

### 4.5. Bacterial Growth Curve Assay

The antibacterial activity of DIMBOA and its derivatives were evaluated by examining the optical density (OD) growth curves. Nine sterilized triangle flasks were prepared for each derivative. CDHB and DIMBOA dissolved in DMSO were added into 100 mL of rich B medium to obtain final concentrations of 200, 100, and 50 mg/L. BOA was adjusted to 300, 200, and 100 mg/L, and MBT had lower concentrations of 50, 25, and 10 mg/L and with 1% DMSO as control. The medium was inoculated with 500 μL of the overnight-cultured bacterial suspension (OD_600_ = 1.0) and incubated at 180 r/min for 24 h at 30 ± 1 °C. Cell density was detected by measuring OD_600_ values at 2-h intervals during the 24-h cultivation. The OD_600_ values were recorded using an Evolution 300 UV-Vis spectrometer (Nicolet, Madison, WI, USA,). Each concentration was determined in triplicate and calculated to obtain the average value.

### 4.6. Biofilm Formation Assay

Crystal violet staining was used to evaluate the film-forming ability of *R. solanacearum* influenced by DIMBOA and its derivatives in polystyrene microtiter plate through a previously reported method with minor modifications [46,47]. Five sterilized centrifuge tubes were prepared for each derivative. Each tube was added with 3 mL of the mixed cultures [15 μL of inoculum (OD_600_ = 1.0) washed twice by sterile distilled water, mixed with final BOA and DIMBOA concentrations of 50, 100, 200, and 300 mg/L in B medium and with 1% DMSO as control]. The concentrations of CDHB and MBT were adjusted to 10, 25, 50, and 100 mg/L. Biofilm growth was initiated by inoculating 200 μL of the mixed cultures into individual wells of a 96-well microtiter plate. The plate was incubated without shaking for 24 h at 30 ± 1 °C. At the endpoint of the incubation period, the medium was removed and immediately washed three times with sterile deionized water. Biofilms of each well were stained with 0.1% crystal violet for 30 min. After staining, the crystal violet was removed and the biofilm was immediately washed three times to remove excess stain. The stained biofilm was dissolved in 200 μL of 95% ethanol and, quantified using absorbance values determined using a microplate reader at OD_490_. Each treatment had six replicates, and the experiment was performed at least three times.

### 4.7. Swarming Motility Assay

Swarming motility assay was performed on semi-solid motility medium as previously reported by Tan [48] with minor modifications. Briefly, B medium supplemented with 0.8% glucose and 0.5% agar was used to assess swarming motility. DIMBOA and its derivatives (50 mg/L) were added to motility agar, with DMSO as control. Plates containing different DIMBOA and derivative concentrations were air dried for 30 min in a laminar flow hood. A double deck sterile filter paper disc (6 mm diameter) was placed in the center of each plate *R. solanacearum* cells at the logarithmic growth phase (OD_600_ = 1.0) were collected from 10-fold diluted B liquid culture and re-suspended in sterile deionized water after gentle spinning at 4000 rpm and 4 °C for 3 min. Bacterial suspensions (5 μL) were loaded on the filter paper discs and drop inoculated at the center of the swarm agar medium plates. After incubation for 24 h at 30 ± 1 °C, colony diameters were measured in three directions on each plate in triplicate. The results are expressed as the mean of three separate assays for each determination.

### 4.8. Greenhouse Experiments

To evaluate the safety of DIMBOA and its derivatives on tobaccos, a pot experiment was carried out in a greenhouse maintained at 28 °C/30 °C (night/daytime temperatures) with 90%–95% relatively humidity. The 10-mL solution of DIMBOA and its derivatives and DMSO (0.3%) were respectively irrigated into the rhizosphere of each 4-week-old tobacco seedling, then the fresh weight was measured after 7 days. This pot experiment included six treatments, BOA at concentrations of 300 mg/L, CDHB at concentrations of 100 mg/L, MBT at concentrations of 50 mg/L, DIMBOA at concentrations of 200 mg/L, DMSO and water control group, respectively. The assays were independently repeated three times. A total of 180 plants were used for evaluation of the toxic of DIMBOA and its derivatives. Each treatment comprised 30 plants. 

Pot experiments were conducted to determine the effect of DIMBOA and its derivatives on tobacco bacterial wilt. Briefly, 10 mL of the solution of DIMBOA and its derivatives (10 mg/L) and DMSO (0.05%) were irrigated into tobacco roots when the fourth or fifth leaf is unfolding. Freshly cultured suspension of *R. solanacearum* (1 × 10^8^ CFU /mL, 10 mL) was inoculated to the rhizosphere of each seedling and treated with DIMBOA and its derivatives after 24 h. The experiments were conducted with 20 replicates and repeated three times under identical conditions. The incidence of tobacco bacterial wilt was investigated every day after root irrigation. The disease index and the control efficiency were calculated by formula [47].

## 5. Conclusions

In conclusion, the principal cyclic hydroxamic acids, 2,4-Dihydroxy-7-methoxy-1,4-benzoxazine-3-one (DIMBOA) was isolated from 7-day-old etiolated maize seedlings and characterized by UV, melting point apparatus, and NMR. Moreover, this is the first to report the anti-*R. solanacearum* activity of the isolated DIMBOA and its derivatives 2-benzoxazolinone (BOA), 6-chloro-2-benzoxazolinone (CDHB), and 2-mercaptobenzothiazole (MBT) against *Ralstonia solanacearum.* MBT showed the strongest antibacterial activity, followed by CDHB and DIMBOA, with minimum inhibitory concentrations (MICs) of 50, 100 and 200 mg/L, respectively, better than the BOA with 300 mg/L. The results showed that hydrosulphonyl and chlorine substituents enhanced the bactericidal activity against *R. solanacearum*. DIMBOA and its derivatives at concentrations of up to 50 mg/L exhibit high anti-biofilm formation activity partially, and also significantly repressed the swarming motility of *R. solanacearum*, resulting in weaker pathogenicity. DIMBOA and its derivatives did not reduce bacterial wilt incidence significantly on a susceptible cultivar Yunyan 87 under greenhouse conditions when inoculum concentrations up to 10^8^ CFU/mL were used in this study. But DIMBOA showed the possibility of elicitors and reduced the disease index of bacterial wilt. The efficacy of DIMBOA and its derivatives in controlling bacterial wilt on susceptible tobacco cultivars under natural field conditions where low inoculum concentrations exist deserves further studies. Furthermore, we could explore the application effect of DIMBOA-induced systemic resistance to bacterial wilt in tobacco.

## Figures and Tables

**Table 1:** Disk diffusion susceptibility testing results for DIMBOA and its derivatives.

Concentration (μg/disc)	Inhibition Zone (mm)			
	BOA	CDHB	MBT	DIMBOA
0	0.0 ± 0.00	0.0 ± 0.00	0.0 ± 0.00	0.0 ± 0.00
10	0.0 ± 0.00 ^a^	2.4 ± 0.22 ^a^	3.5 ± 0.29 ^a^	1.5 ± 0.29 ^a^
50	1.2 ± 0.17 ^a^	4.3 ± 0.33 ^a^	7.0 ± 0.58 ^ab^	4.5 ± 0.29 ^b^
100	5.0 ± 0.58 ^b^	10.5 ± 0.50 ^b^	8.0 ± 0.58 ^b^	4.5 ± 0.29 ^b^
200	9.67 ± 0.83 ^c^	17.7 ± 0.44 ^c^	11.0 ± 0.50 ^b^	13.7 ± 0.33 ^c^
300	10.83 ± 0.93 ^c^	22.0 ± 1.26 ^d^	17.7 ± 2.73 ^c^	19.3 ± 0.33 ^d^

Each experiment was repeated in three times. Lower case letters indicate significant differences according to Duncan’s test (*p* < 0.05).

**Table 2:** The minimum inhibitory concentrations (MICs) and minimum bactericidal concentrations (MBCs) of DIMBOA and its derivatives against *R. solanacearum* in the 96-well polystyrene microtiter plates.

DIMBOA and Its Derivatives	MIC (mg/L)	MBC (mg/L)
BOA	300	>1000
CDHB	100	500
MBT	50	400
DIMBOA	200	500

**Table 3:** IC_50_ and IC_90_ for DIMBOA and its derivatives against *R. solanacearum.*

DIMBOA and Its Derivatives	Regression Equations	IC_50_ (mg/ L)		IC_90_ (mg/L)	R Value
BOA	*Y* = 2.0144*x* + 0.3265	208.92	904.05		0.9938
CDHB	*Y* = 1.0285*x* + 3.4861	29.65	522.50		0.9733
MBT	*Y* = 0.9786*x* + 4.1034	8.25	168.18		0.8930
DIMBOA	*Y* = 1.6101*x* + 2.1541	58.55	366.03		0.9015
